# Chorea in a Horse

**Published:** 1883-07

**Authors:** John Lindsay

**Affiliations:** Huntington, L. I.


### XV.—CHOREA IN A HORSE.

REPORTED BY JOHN LINDSAY, D.V.S.

April 25th, 1883.

Was called to attend a horse suffering from stiffness of the posterior extremities. When compelled to move, the legs were carried like a couple of sticks, there being no apparent movements at the joints. The temperature was 102° F., pulse 48, membranes of the eyes and nose injected, respirations normal, eyes dull, and head dependent.

My diagnosis was spinitis from over stimulation, as I learned from the owner that the animal had recently been changed from six to twelve quarts of oats per day, and at the same time had been allowed to remain unused in the stable for one week that he might make a good appearance for sale.

I ordered all grain stopped and placed him upon bran mashes, enemas and hot applications to loins.

26th.—Stiffness somewhat less; temperature normal; pulse normal; mucous membranes clear; appetite and thirst normal. Gave a pill containing full dose of aloes and calomel.

27th.—The cathartic given the day before worked nicely. The stiffness all disappeared; urine now clear as water; caught some, and upon examining it found that it contained sugar; thirst greatly increased; appetite good; the left leg was automatically thrown against the side of stall with considerable force. I now ordered kali bromidium ℥ss to be given three times a day.

28th.—The automatic movements have changed from the left side to the right and are as violent as those on the left side yesterday. Treatment continued.

29th.—The automatic movements have stopped while the animal stands quiet, but commences again as soon as the animal attempts to move, although very moderate.

30th.—The animal now moves freely and naturally; thirst normal; urine of a pale, sherry color; appetite good. The patient discharged, cured.

In this connection I would say that I have seen a number of similar attacks in pigs and they have all recovered upon the bromide treatment after free catharsis.

HUNTINGTON, L. I., May, 1883.

